# Toll-like Receptors and Inflammatory Bowel Disease

**DOI:** 10.3389/fimmu.2018.00072

**Published:** 2018-01-30

**Authors:** Yue Lu, Xinrui Li, Shanshan Liu, Yifan Zhang, Dekai Zhang

**Affiliations:** ^1^Department of Gastroenterology and Hepatology, The Second Affiliated Hospital of Harbin Medical University, Harbin, Heilongjiang, China; ^2^Center for Infectious and Inflammation Diseases, Texas A&M University, Houston, TX, United States

**Keywords:** inflammatory bowel disease, toll-like receptors, innate immunity, immune dysfunction, fecal microbiota transplantation

## Abstract

Inflammatory bowel disease (IBD) is one relapsing and lifelong disease that affects millions of patients worldwide. Increasing evidence has recently highlighted immune-system dysfunction, especially toll-like receptors (TLRs)-mediated innate immune dysfunction, as central players in the pathogenesis of IBD. TLRs and TLR-activated signaling pathways are involved not only in the pathogenesis but also in the efficacy of treatment of IBD. By understanding these molecular mechanisms, we might develop a strategy for relieving the experience of long-lasting suffering of those patients and improving their quality of life. The purpose of this review article is to summarize the potential mechanisms of TLR signaling pathways in IBD and the novel potential therapeutic strategies against IBD.

## Introduction

**Figure 1:**
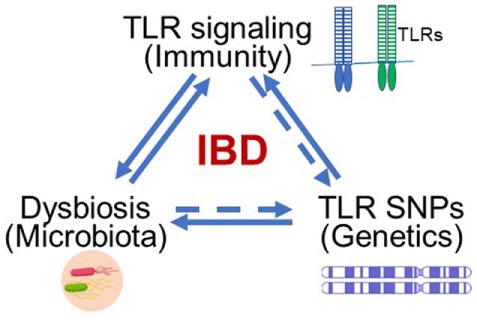
Toll-like receptors (TLRs) serve as the hub of immune responses to microbes in the gut in inflammatory bowel disease (IBD) pathogenesis. The abnormal TLR signaling may trigger disease-related inflammation. TLRs are key sensors in the gut to recognize abnormal intestinal microbes to induce immune response and inflammatory disease. Genetically polymorphisms such as single-nucleotide polymorphism (SNP) may alter the composition of microbiota in the gut.

Inflammatory bowel disease (IBD), including Crohn’s disease (CD) and ulcerative colitis (UC), has become a global health burden with increasing incidence and prevalence (1). Although the exact etiology of IBD remains unclear, we have long recognized the close relationship between IBD and such factors as immunity (2–4), environment (5), genetics (6), and diet (7). Among these factors, cellular receptors in the innate immune system are fundamental and recognize pathogenic molecules to trigger immune responses. Additionally, some toll-like receptor (TLR) polymorphisms/mutations have been identified and directly linked to IBD. Genetic alterations of these receptors might change the composition of microbiota in the gut. Therefore, receptors of the innate immune system, such as TLRs, impact many aspects of IBD etiology, including immune responses, genetics, and microbiota (Figure 1).

As is well acknowledged, IBD results from an inappropriate response of a dysfunctional mucosal immune system to the resident microbiota and other noxious antigens. In intestinal ecology, trillions of indigenous commensal microorganisms, including bacteria, fungi, and viruses (8), maintain homeostasis along with the elements of the host immune system (9). However, the introduction of an innate immune defect, for example, epithelial barrier leak and/or mucosal destruction can harm the otherwise beneficial host–microbe balance. Furthermore, the intestine’s access to an outside environment facilitates invasion by extrinsic pathogens, thereby inciting a host immune response to contribute to diseases.

Recently, we realized that TLRs serve as the hub of immune responses to microbes in the gut, thereby inciting IBD (Figure 1). The related research led to this summary of advances in current studies about the relationship between TLR signaling pathways and IBD and our perspective about potential therapeutic strategies for alleviating IBD.

## TLRs are Key Immune Sensors of Microbiota in the Gut

As the body’s initial defense weapon, innate immunity plays a critical role in recognizing pathogens and maintaining intestinal homeostasis. TLRs are pattern recognition receptors that work as the immune system’s protective sentries whose job is to sense and recognize pathogen-associated molecular patterns (PAMPs). PAMPs are highly conserved structures of microbes, such as unmethylated double-stranded DNA, single-stranded RNA, lipopolysaccharide (LPS), lipoproteins, and flagellin (10). Upon activation, TLRs become dimerized and trigger the subsequent activation of downstream signaling cascades, such as inducing a variety of inflammatory cytokines through transcription by mediating the phosphorylation of IκB to activate NF-κB. Furthermore, TLR activation regulates the maturation of dendritic cells (DCs) and induces the proliferation and differentiation of Th1 and Th2 T cells.

Because the TLR signaling involves in many life-threatening diseases, TLRs have been the subject of intense study (11–14). IBD is only one of these devastating conditions; nevertheless, such patients exhibit far different expressions of TLRs in comparison with healthy controls. Most TLR signaling pathways participate in the progression of IBD, performing sometimes beneficial and other times harmful functions. As an essential adaptor of TLR pathways, myeloid differentiation primary response 88 (MyD88) is a proven actor in the breakdown of immune tolerance. As mentioned above, TLRs not only control innate immunity but also critically regulate adaptive immunity, such as T cell activation. By restraining the TLR-induced over immune responses, T regulatory cells (Tregs) inhibit other T cells from functioning effectively to maintain immune tolerance and have a key role in promoting tolerance at host–microbial interfaces (15). The balance between Tregs and effector T cells is disturbed in patients with IBD. That is, when Tregs’ function of providing immune tolerance is suppressed and effector T cells like Th1, Th2, Th17, and NKT cells are activated, producing and releasing inflammatory cytokines, the progression of an inflammatory disease like IBD grows out of control (3, 16).

## Gut Homeostasis is Maintained by the Mucosal Immune Response and the Microbiota

The composition and amount of human gut microbiota change with age. During different stages of human growth and development, the gut flora becomes stabilized and gradually specialized which is individually identifiable. Consequently, in children, the gut microbiota is much less diverse than that in adults (17).

To maintain the gut’s homeostasis, interaction between the host’s immune system and intestinal commensal microbiota is crucial. It is not surprising that antibiotics or antiviral treatment that affect the commensal microbiota result in the fact that the intestine would be more susceptible to pathogens and diseases (18, 19), including IBD. Resident microbiota not only fosters normal development of the immune system but also makes immunity work to maintain intestinal health and provide signals that influence the ensuing immune responses. Alternatively, defects in the innate immune system can lead to dysbiosis of the intestinal microbiota, leading to the host metabolic disorder. Ultimately, innate immunity has a role in determining the composition of each individual’s microbiota (8). Interactions between the intestine’s commensal microbiome and host immunity are mediated, at least in part, through TLRs.

## TLRs are a Potential Molecular Mechanism of IBD

Toll-like receptors, as sensors of gut microbiota, play a critical role in maintaining the gut’s homeostasis, controlling the immune responses and shaping the microbiota. In a mouse model, Inoue et al. found that the postnatal expression of TLR2 and TLR4 in intestinal epithelial cells (IECs) is dynamic and depends on the presence of commensal intestinal microbiota (20). In reporting about the relationship between intestinal TLRs and commensal microbiota, others showed that oral antibiotic treatment resulted in the upregulation of TLR4, TLR5, and TLR9 in the ileum and TLR3, TLR4, TLR6, TLR7, and TLR8 in the colon; meanwhile the expression of TLR2, TLR3, and TLR6 in the ileum as well as TLR2 and TLR9 in the colon diminished (20, 21). Additionally, the diversity and total amount of microbiota decreased. Those results confirmed that the microbiota could regulate TLR expression (21). Although TLRs’ ability to exert a protective effect against IBD has been noted, controversy remains (22). Thus, the actual contribution of TLRs to inflammation and commensal dysbiosis remains uncertain (23), although the activation of TLR signaling triggers a serial cascade of downstream events that clearly play an important role in the development of IBD.

**Figure 2:**
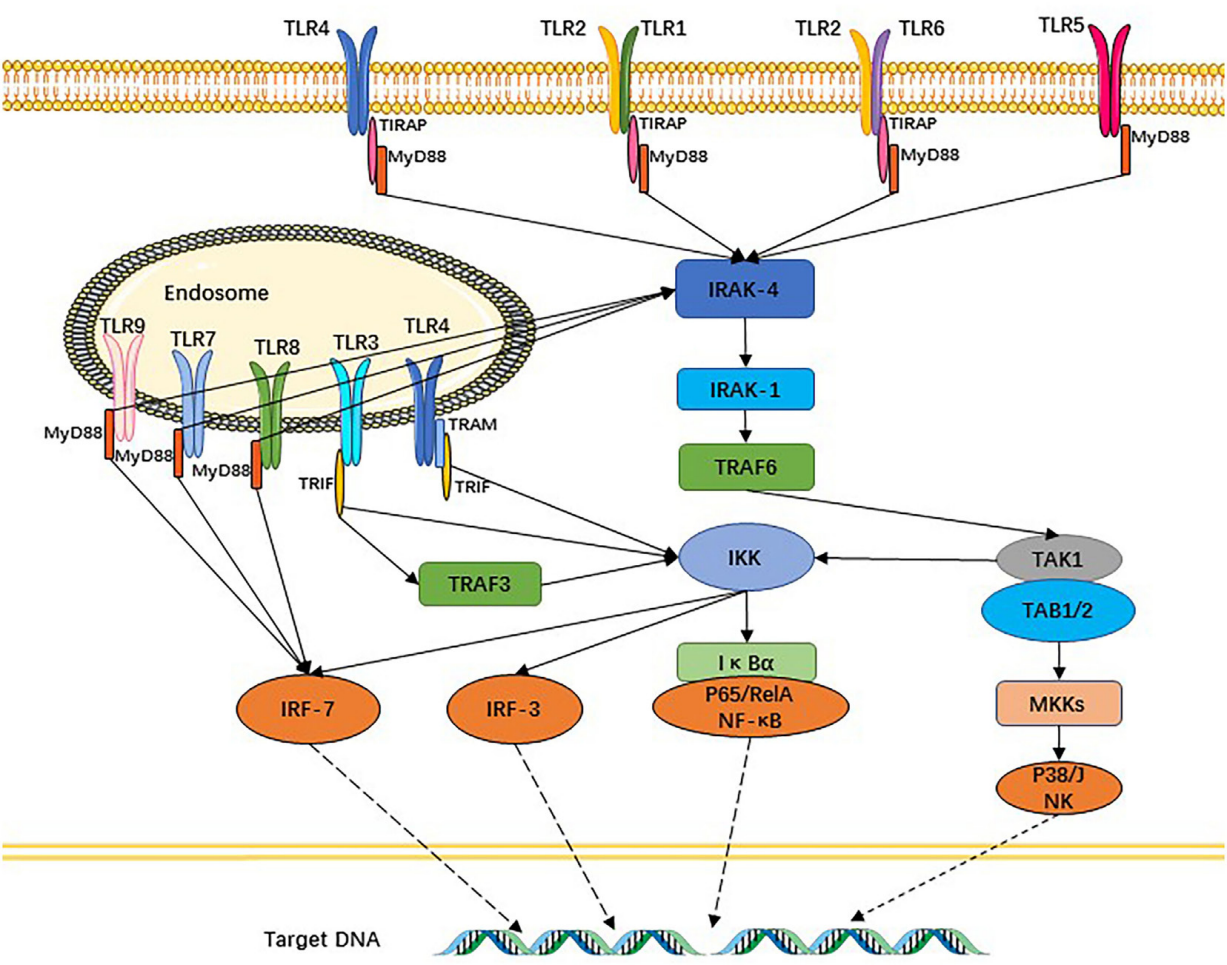
Toll-like receptors (TLRs) and their signaling pathways. TLR1 and TLR6 recognize their ligands as heterodimers with TLR2. For TLR4, MD2, and CD14 are required for lipopolysaccharide recognition and signaling. TLR3, TLR4, TLR5, TLR7, and TLR9 are currently thought to deliver their signal by forming homodimers after interacting with their ligands. TLR3, TLR7/8, and TLR9 are intracellular TLRs and recognize nucleic acids.

The TLR signaling pathway is very similar with the interleukin (IL)-1R family, characterized by the requirement for a Toll-IL 1 receptor (TIR) domain-containing adaptor protein (TIRAP), protein kinase, and a transcriptional factor to transfer the signal. Except for TLR3, other TLR signaling pathways depend on MyD88 to activate NF-κB and MAPK (mitogen-activated protein kinase) to control the inflammatory response. The domain-containing TIRAP lying downstream of TLR1, 2, 4, and 6 recruits MyD88. Meanwhile, TRIF (TIR domain-containing adaptor inducing IFNβ) participates in TLR3 and TLR4 signaling pathways. Thus, TLR signaling is divided into two types of pathways: one of which is MyD88-dependent and the other MyD88-independent but TRIF-dependent. Downstream of the TLR signaling pathways, activated NF-κB and IRF [interferon (IFN) regulatory factor] control their target genes (10, 24) to produce an abundance of inflammatory cytokines and IFNs, which improve resistance to and clearance of pathogens from the body, and can also promote inflammation (Figure 2). Other functions of the latter factors are to upregulate the expression of related genes responsible for phagocytosis and possessing the ability to enhance phagocytic function and to kill microbes (25). Apart from these functions, TLR signaling recruits activated natural killer cells (NK cells) and DCs (26, 27). DCs are prompted by TLRs to present antigens to T cells and initiate T cell responses, thus providing a bridge between innate immunity and adaptive immunity (28, 29). TLRs and the signaling pathway also exist in T cells and, once intrinsic TLR signaling in T cells is lost, it results in significant changes in the gut’s microbial composition. Follicular helper T cells are abundantly produced in germinal center and interact with B cells, a process that is also mediated by T cell-intrinsic TLR signaling (30). TLRs additionally regulate B-cell responses for the purpose of producing monospecific IgM, IgG, and IgA antibodies (31), which involved in adaptive immunity that can mediate intestinal homeostasis and regulate microbiota content.

## TLRs are Associated with IBD

Toll-like receptors have been detected in both IECs and stromal tissue cells of the gastrointestinal tract (Table 1), and TLR1, 2, 3, 4, 5, and 9 are expressed in the small and large intestines of mice and humans (32). However, TLR6, 7, and 8 are present only in the human colon and murine small intestine (32). With regard to patients suffering UC, though, individual TLRs are differentially expressed in the intestinal epithelium (33). That is, the expression of TLR2, 4, 8, and 9 genes are upregulated in patients with active UC, whereas TLR5 tends to be upregulated in those with active UC (compared to quiescent UC disease) and downregulated in quiescent UC (compared to controls with healthy colonic mucosa) (34) (Table 1).

**Table 1:** The role of toll-like receptors (TLRs) and their activated signaling in inflammatory bowel disease (IBD).

TLRs	The role in IBD	Reference
TLR1/2	Preventing chronic inflammation	(38, 39)
TLR2/6	Promoting colitis	(25, 36)
	Suppressing immune response	(37)
TLR3	Promoting protective immunity under inflammation conditions	(55)
TLR4	Causing intestinal tissue destruction and ulceration	(40–42)
	Protective function	(43)
TLR5	Preventing diseases associated with intestinal inflammation	(52)
TLR7	Promoting protective immunity under inflammation conditions	(55)
TLR8	Inducing mucosal inflammation	(56)
TLR9	Protective function	(58–60)

### TLR1, TLR2, and TLR6

TLR2 must form heterodimers with TLR1 or TLR6 to transmit a signal. The TLR2/6 heterodimer recognizes diacylated lipopeptides, whereas TLR1/2 can recognize triacylated lipopeptides (23). The inhibition of TLR2/6 signaling has played a beneficial role by slowing down IBD progression. When TLR2-p, a TLR2 transmembrane domain-derived peptide, interacted directly with TLR2 within the membrane, thereby inhibiting TLR2–TLR6/1 activity, the result was significantly ameliorated dextran sodium sulfate (DSS)-induced colitis, as assessed by histology, colonoscopy, and body weight measurements (35). Further, Depaolo et al. demonstrated that TLR2/6 stimulation of DCs, both *in vitro* and *in vivo*, was associated with the formation of suppressive immune responses (36). However, another group reported that TLR6 was overexpressed in the intestines of IBD patients and might promote experimental colitis in mice (23). They also pointed out that TLR6 was an important driver of Th1 and Th17 responses (23). This conclusion might indicate a potential target for future IBD treatment, since TLR6-deficient mice manifest decreased Th1 and Th17 responses in gut-associated lymphoid tissue compared to their TLR6-sufficient counterparts (23). Although the function of TLR2/6 may be bidirectional, the particular mechanism remains unclear.

Previous study confirmed that the TLR1 signaling pathway was crucial for mucosal protection against oral infection caused by a *Gram*-negative pathogen (37). More multifaceted results suggested that the absence of TLR1 during acute gastrointestinal infection led to chronic immune activation and transmutation in the composition of the commensal bacteria (38). Overall, this background indicates that TLR1 and its signaling pathway might prevent chronic inflammation of the colon in IBD.

### TLR4

TLR4 is the first identified TLR in mammalian system and recognizes LPS in Gram-negative bacteria. Under physiologic conditions, TLR4 is expressed at a low level in IECs (39). Although the primary role of TLR4 is beneficial for induction of an inflammatory response providing protection from invading bacteria and promoting mucosal integrity; in some experiments, TLR4 proved to be maladaptive, actually causing tissue destruction and ulceration (39). Considering that TLR4 gene expression was upregulated in the intestinal epithelia of patients with active UC, TLR4 might be a participant in UC disease development. One study showed that stimulated enteric glial cells (EGCs) could release nitric oxide *via* TLR4, leading to the liberation of pro-inflammatory cytokines, which would aggravate gut inflammation (40). Elsewhere, LPS (a TLR4 ligand) stimulation of monocytes and conventional DCs elicited high levels of pro-inflammatory cytokines, which would intensify the DSS-induced colitis (18). Afterward, a nutritional activator of innate immunity, wheat amylase-trypsin inhibitor (ATI), was found capable of interacting with TLR4 on myeloid cells (41). Oral ATIs then proved to induce intestinal myeloid cell infiltration and activation as well as release of inflammatory mediators in the colon, mostly through the TLR4 pathway (42). The foregoing conclusions had important implications for the onset and severity of inflammatory intestinal disease (42). Much evidence supports the theory that the TLR4 signaling pathway has a negative role in IBD. Interestingly, Gibson et al. obtained experimental results denoting that TLR2 could protect against many noxious agents. In addition, TLR2 cyto-protective responses from tissue-resident cells maintained mucosal integrity against the lethal TLR4-dependent inflammatory responses of hematopoietic cells. Thus, the role of TLR4 during colitis could be either protective or damaging (43).

### TLR5

For TLR5, the relationship is closely linked to IBD. As a kind of transmembrane innate receptor, TLR5 distribution is polarized; in the colon, it is expressed on basolateral surfaces of gut epithelial cells, whereas in the ileum its expression is both apical and basolateral (44, 45). Mainly, TLR5 recognizes bacterial flagellin, polymerizing monomer subunits making up flagella, the whip-like structures providing motility for such bacteria as *Salmonella* and *E. coli* (46, 47). Systemic immunization with sFliC, a flagellin expressed in *Salmonella typhimurium*, induces the recruitment of CD103^+^ DCs from the lamina propria to the mesenteric lymph nodes in a TLR5-dependent manner (48). CD103^+^CD11b^+^LPDCs (lamina propria DCs), as the primary TLR5-expressing cells in the small intestine, expressed large amounts of IL-23 following bacterial flagellin administration and produce RegIIIγ in an IL-22-dependent manner (49). Through polymerase chain reaction-restriction fragment length polymorphism and sequencing, Meena et al. found that polymorphisms in the TLR5 genes, *R392X* and *N592S*, were significantly associated with the UC in American and Indian populations (50). Additionally, the cytokine level was significantly modulated in patients with different genotypes of TLR4 and TLR5 single-nucleotide polymorphisms (50). A non-synonymous coding variant (rs5744174) in the TLR5 gene also exhibited a modest association with CD in children (51). A possibly related clue is that TLR5-deficient mice have an altered composition of intestinal microbiota compared with wild-type mice, develop only moderate protection from inflammation, and are susceptible to colitis. To summarize, the expression of TLR5 on IECs not only regulates the composition and localization of intestinal microbiota but also prevents diseases associated with intestinal inflammation (52). A consequence of inhibiting TLR5 activation might reduce the production of antimicrobial peptides in intestinal epithelium cells (53). Overall, TLR5 appears to play a critical in the development of IBD, and may be a good target for developing a promising therapeutic strategy against IBD.

### TLR3, TLR7, and TLR8

TLR3 has been described as significantly downregulated in epithelial cells of patients with active IBD (54). However, another study of humans with IBD showed that combined TLR3 and TLR7 genetic variations significantly influenced the severity of UC, which led to higher cumulative hospitalization rates. This report also stated that TLR3 and TLR7 agonists stimulated IFN-γ secretion by pDCs thereby promoting protective immunity under inflammatory conditions (18). Still others presented data that TLR9, TLR3, or TLR7 agonists could induce type I IFN, which can prevent experimental colitis (55).

As previously established, TLR8 is upregulated in patients with active UC; however, less is known about the mechanism of TLR8 in disease activity. TLR8 signaling enhanced generation of tumor necrosis factor (TNF)-α and IL-1β, both of which have been associated with mucosal inflammation in UC. Interestingly, in both common predisposing and protecting haplotypes, TLR8 is an X-linked IBD susceptibility gene. These findings suggest the importance of genetic variation in innate immunity as a crucial factor of UC (56).

### TLR9

TLR9 lying on intracellular endosomes recognizes bacterial CpG DNA (57). In UC patients, the severity of endoscopic and histological inflammation correlated positively with TLR9 expression (34). TLR9 activation has been shown to prevent the development of mucosal inflammation and promote wound healing in several colitis models (58, 59). After activation of the TLR9 signaling pathway by its agonist, significant improvements were found with respect to clinical remission of UC with mucosal healing and early reduction of symptoms (60). Thus, the protective function of TLR9 is relatively clear.

## Effector Factors Downstream of TLR Signaling Pathways in IBD

The TLRs lying upstream of the signaling pathways seem like the scouts, whereas infantries of the army are the effective cytokines secreted by target cells, such as epithelial cells, DCs, and T cells. In this section, we will analyze and discuss the roles of effectors involved in the TLR signaling pathways, whether beneficial or noxious (Figure 2).

### Antimicrobial Non-Defensin-Family Proteins

Toll-like receptors have the ability to promote IECs and Paneth cells to produce RegIIIβ/γ (19), a type of antimicrobial non-defensin-family proteins that can kill Gram-positive bacteria. Cash et al. demonstrated that, at mucosal surfaces, RegIIIβ/γ expression would be triggered by increased microbial–epithelial contact. Increased expression of Reg proteins in IBD patients may be a compensatory response that limits mucosal penetration by gut microbes (61). There is no doubt that Reg proteins and the arousal of activated TLR signaling pathways have a definite protective function for intestines in defending against the invasion of pathogens.

### Interferons

Interferons are cytokines involved in the effective function of TLR signaling pathways. As mentioned previously, an article published in the journal of *Cell* clearly indicated that resident viruses elicit protective immunity through TLR3 and TLR7-mediated IFN-β by DCs in the inflamed gut (18). IFNs-α/β play a key role in regulating the innate immune system, especially by modulating the functions of macrophages and DCs (62). For example, IFN-β induces a clinical response and remission in a large population of patients with UC (63). Thus, type I IFNs, including IFNα and IFNβ, perform a potentially important protective role in intestinal homeostasis. This scenario suggests that strategies to modulate innate immunity may have therapeutic value for ameliorating intestinal inflammatory conditions (64). Katakura et al. similarly indicated that IFN-α/β inhibited the severity of DSS-induced colitis *via* suppressing macrophage pro-inflammatory signaling (65). A similar conclusion came from a survey analyzing CD103^+^CD11b^−^ DCs as the fundamental regulators of intestinal homeostasis through regulating IFN-β-induced anti-inflammatory proteins in IECs (66). These results have repeatedly emphasized the helpful effect and important role of IFNs and innate immunity in controlling the pathogenesis and progression of IBD.

### Interleukin

Interleukin is a part of a large family whose members have diverse and complex functions. As an effective cytokine involved in the TLR signaling pathway, IL plays bidirectional roles in the pathogenesis and progression of IBD. In human, both TLRs and IL-1Rs have a TIR domain and are considered as a super family. IL-1 family members can also perform opposing roles in gut health and disease, espousing a novel pathogenic hypothesis to account for their abilities. IL subtypes have important translational implications concerning the prevention and treatment of chronic intestinal inflammation, including CD, UC, and CRC (colorectal adenocarcinoma) (67). For example, IL-18 inhibits goblet cell maturation by regulating the transcriptional mechanisms that controls goblet cell development. These results reveal that goblet cell dysfunction might contribute to the underlying pathology of UC (68). In the IL-1R signaling pathway, which is similar to TLR signaling pathways, the IL-1R complex can recruit the adaptors and MyD88 to the TIR domain to trigger downstream cascades. After several kinases are phosphorylated, and then NF-κB is translocated into nuclei of the cells, such as macrophages and DCs, and the inflammatory genes are expressed (59).

Other than IL-1, other ILs are involved in IBDs. That is, some evidence indicates that certain ILs provide protective actions in the pathogenesis of IBD. When the TLR7/IL-22 pathway is controlled, the restoration of immune-mediated colonization resistance follows, and infection by intestinal pathogens is limited upon antibiotic exposure (69). Tosiek et al.’s experimental findings indicated a potentially beneficial role of IL-15 in IBD by subtly modulating the balance between Tregs and Th17 cells and controlling intestinal inflammation (70). Kim et al. then found that, after selectively impairing TLR4-mediated IL-12 production and the host defense response, the major CD-associated NOD2 mutations could cause a primarily immune-deficiency phenotype CD (71). Therapeutics that compromise innate immune responses (TLR2 signaling, in particular) may not be beneficial to patients with colitis and can even worsen symptoms. However, treatments that stimulate cytokines’ protective responses, like IL-11, could benefit patients with colitis (43). A related study confirmed that TLR5 on bone marrow-derived cells activates a signaling pathway that makes Th17 lymphocytes produce the IL-17 and IL-22 cytokines thought to activate protective pathways in epithelial cells (72).

Conversely, some ILs may promote inflammation and hasten the progress of IBD. For example, IL-6 increased pathogenic cytokine production by intestinal intrinsic lymphocytes in chronic intestinal inflammation, indicating that this pathway may be regulated in IBD. This finding extended the idea that therapeutic strategies targeting intrinsic lymphocytes or their proximal cytokine signals may offer a new treatment plan for IBD (73). Stimulation with LTA (TLR2 ligand) or LPS resulted in activating the TLR signaling pathway, then promoted the progression of NF-κB p65 nuclear translocation and significantly increased IL-8 secretion. Intestinal myofibroblasts expressing TLRs are activated directly by bacterial components and are known to respond to inflammatory signals, therefore, may play a role in CD-associated fibrosis (74).

Above all, we can see that no matter whether the function of ILs is protective or noxious, they play an important role in IBD. In either case, ILs provide novel therapeutic strategies.

### Tumor Necrosis Factor-α

Tumor necrosis factor-α is a main cytokine in systemic inflammation and is a contributor to acute reactions produced mainly by activated macrophages. TNF-α, a pro-inflammatory cytokine, contributes to the injury too (75). Pelczar et al. suggested that anti-TNF-α therapy might work in part by suppressing IL-22BP and could provide a promising treatment for IBD (76). It has been confirmed that macrophages from patients with CD have attenuated the release of TNF-α downstream of multiple TLRs. Abnormal TNF-α secretion from downstream of multiple TLRs, was found to affect all disease phenotypes but unrelated to polymorphisms associated with CD. Impairment of TNF-α release is particularly severe in patients with colonic CD (77). TNFR2 has the capacity to promote the apoptosis of colonic CD8^+^ T cells; moreover, in that circumstance, CD8^+^ T cells are necessary to promote colitis. Conjecturally, if TNFR2 signaling was disrupted, the outcome might attenuate the pathogenesis of CD (78).

## The Significance of TLRs in Clinical Treatment of IBD

The goals for treatment of patients with IBD are to improve quality of life and to prevent complications. Medications routinely prescribed for these patients include aminosalicylates, corticosteroids and such immunosuppressive agents as natalizumab and vedolizumab. Relevant experiments have provided statistically significant data indicating improved mucosal healing and symptomatic remission in UC patients after the activation of TLR9 (60). Since then, medications targeting TLRs and the TLR signaling pathway have been developed that predict a promising future for alleviating the symptoms of IBD.

A range of immunologic results has produced the following sequence of activities with possible relevance to IBD therapies (79). Nur77, an intracellular transcription factor and mediator of inflammatory responses, has been confirmed as an important regulator of TRAF6/TLR-IL-1R-initiated inflammatory signaling. A loss of Nur77 would promote the development of IBD (80). The outcome of experiments with Nur77 suggested its use for the prevention and treatment of IBD (80). In DCs, the Pam3 (Pam3CSK4)-TLR2 axis interfered with the binding of TLR9-induced IFN regulatory factor 5 to the IL12b promoter. In turn, ERK2 (extracellular signal-regulated kinase 2) activation by TLR2 was found to suppress TLR9-induced IL12b gene expression after stimulation by heat-killed *Brucella abortus* (81). *Via* this mechanism, expression of the pro-inflammatory cytokines IL-12/23p40, IL-12, and IL-23 undergo interference. Such suppression of TLR cross talk may prevent excess inflammatory responses to commensal microbes in the gut and, thereby, act as therapeutic strategies for alleviating intestinal inflammatory and autoimmune diseases (82).

In the TLR family, TLR2 and its signaling pathway are promising therapeutic targets. As previously mentioned, the therapeutic implication of TLR2-p was investigated using the DSS colitis model. TLR2-p treatment significantly ameliorated colitis-associated conditions (83). Shmuel-Galia et al. showed that the TLR2-p was co-localized with TLR2, and physically interacted with TLR2, TLR1, and TLR6 (35). They found that TLR2-p inhibited lipoteichoic acid-induced TLR2–TLR6 assembly, thus inhibiting downstream signaling, such as ERK phosphorylation. TLR2-p inhibited induction of the pro-inflammatory cytokines IL-6, IL-12, and IL-1β that were crucial in colitis development. Additionally, TLR2-p reduced IL-23 and IFN expression (35). Therefore, an agonist of TLR2 may constitute a treatment method for some diseases of the immune system, including IBD.

Esposito et al. proposed that palmitoylethanolammide (PEA) might act as a new drug to control the acute phase of intestinal inflammation that occurs in UC. Profoundly and beneficially, PEA impacted the abnormal activation of EGCs, mainly *via* peroxisome proliferator-activated receptors γ and inhibited the S100B/TLR4 axis (40, 84).

Fecal microbiota transplantation (FMT), as an ancient method of treating severe diarrhea and food poisoning, was first found in China in the fourth century. After a long quiescence, the potential functions of FMT in recurrent *Clostridium difficile* infection and pseudomembranous colitis have attracted increased attention from clinical doctors and medical scientists. Beyond *C. difficile* infection, other diseases may be ameliorated by using the FMT method (85), Likely examples are irritable bowel syndrome (86, 87) and even such non-gastrointestinal diseases as hepatic encephalopathy (88). However, the mechanism underlying the FMT for treating intestinal diseases has not been investigated thoroughly. Nevertheless, many gastroenterologists have become interested in the application of FMT for intractable IBD.

A multiplicity of studies has proven that the components of microbiota in patients with UC or CD have obvious differences from those of healthy controls (89). Interestingly, though, after FMT, the composition of microbiota from such patients is much more similar to those of healthy donors, and the formers’ symptoms have found relief. Even so, the effectiveness of FMT relies strongly on properties of the donors’ transfer material as well as other factors (90). However, there is no definite standard for the selection of microbiota donors. As mentioned above, microorganism content is strongly related to intestinal homeostasis, and detection of commensal microbiota by the TLR–MyD88 signaling pathway triggers immune responses that are responsible for maintaining host–microbial homeostasis (8). FMT, as a means of microbiota therapeutics, still poses problems to be solved. For example, does transplanted normal microbiota work on the inflamed intestine through an active TLR signaling pathway, or are other mechanisms involved? Which specific components of the whole microbiota provide the protective component(s) and, once identified, is their extraction possible and usable to produce biologic compounds to treat IBD and other diseases? Does FMT have potential side effects? Despite the many problems to be solved, FMT has huge potential to become a highly effective treatment method for IBD and other diseases.

## Concluding Remarks

The immune-pathogenesis of IBD has been a hot topic for study in recent years. As the research progresses, the importance of TLR signaling pathways and innate immunity in the generation of IBD has become a flash point. TLRs and some components of the signaling pathway will turn out to be targets, and both agonists and antagonists can be applied in therapeutic applications. However, only a few TLR agonists and antagonists have advanced to clinical trials. In fact, most members of the TLR family are involved in the progression of this disease. In addition to recognizing the functions of TLR, the direct effectors such as RegIIIβ/γ and IL cytokines as the signaling pathways unfold has become clear. Currently, treatments for IBD that target TLRs have drawn the attention of clinical physicians and research scientists. Although treatment strategies related to TLR are not yet applied or not available in the clinic, we believe that their development will eventually help to resolve the painful disease status of IBD patients.

## Author Contributions

YL performed literature searches, and drafted the manuscript. XL helped edit the manuscript and draw figures. SL and YZ helped write and edit every component of this submission. Dr. D. Zhang developed the idea for this article, helped write, and edit each component of this submission. All authors have approved the final version of this submission.

## Conflict of Interest Statement

The authors declare that the research was conducted in the absence of any commercial or financial relationships that could be construed as a potential conflict of interest.
